# Single-cell analysis of yeast surface display for designer cellulosome applications using a fluorescent protein complex

**DOI:** 10.1128/spectrum.00750-25

**Published:** 2025-08-12

**Authors:** Babette Lamote, Emma Cremelie, Kristel Demeyere, Philippe De Groote, Dennis Grimon, Julie Vanderstraeten, Evelyne Meyer, Marjan De Mey, Yves Briers

**Affiliations:** 1 Laboratory of Applied Biotechnology, Department of Biotechnology, Ghent University 26656, Ghent, Belgium; 2 Centre for Synthetic Biology, Department of Biotechnology, Ghent University 26656, Ghent, Belgium; 3 Laboratory of Biochemistry, Department of Veterinary and Biosciences, Ghent University 703924, Merelbeke, Belgium; Ocean University of China, Qingdao, China

**Keywords:** designer cellulosomes, *Saccharomyces cerevisiae*, yeast surface display, flow cytometry, confocal microscopy, fluorescent proteins

## Abstract

Designer cellulosomes (DCs) are precisely engineered multi-enzyme complexes aimed at lignocellulose saccharification. Achieving spontaneous designer cellulosome display on yeast cell surfaces has been a long-term objective to enhance consolidated bioprocesses with concurrent ethanol production. A “self-assembly” approach involves simultaneous scaffoldin display and docking enzyme secretion. However, challenges arise from the size and complexity of designer cellulosomes, coupled with the yeast cells’ limited capacity for heterologous protein expression. A comprehensive examination of *Saccharomyces cerevisiae* as a host for DC expression remains unaddressed. We meticulously examined the capability of *S. cerevisiae* to produce and display a fluorescent protein complex, which mimics the designer cellulosome architecture and allows for convenient detection of all individual components on the cell surface, using flow cytometry and confocal microscopy. Population-wide analysis revealed a fluorescent protein complex production efficiency of approximately 10%. Single-cell analysis highlighted a clear mutual influence between the expression of scaffoldin and docking proteins, impacting cellular fitness. Newly emerging buds were identified as hotspots for scaffoldin display. The finite capacity of yeast cells to produce heterologous proteins was identified as a major bottleneck. While distributing the cellular load among multiple hosts within a synthetic yeast consortium can alleviate this burden, the use of fluorescent protein complex surface display has visualized the heterogeneity and constraints of *S. cerevisiae* as a host for designer cellulosome expression at the population and single cell level. This study provides a realistic assessment of the challenges in achieving efficient *S. cerevisiae*-based DC display for consolidated bioprocessing.

**IMPORTANCE:**

Efficient and economically viable biomass conversion into fermentable sugars is a pivotal challenge in transitioning from a petroleum-based economy to a bio-economy. Drawing inspiration from nature, cellulosomes represent an exemplary solution for the effective digestion of lignocellulose. These multi-enzyme complexes can be precisely engineered to tailor their properties and transferred to the surface of yeast cells, which can subsequently ferment the sugars into bulk or fine chemicals. Achieving this transfer successfully necessitates a comprehensive understanding of how yeast cells can recombinantly produce and attach such multi-component complexes to their surface. This study employs a fluorescent surrogate to provide novel insights into the capabilities of yeast cells at both the single-cell and population levels.

## INTRODUCTION

The urgent demand for green and sustainable methods to convert renewable biomass into fuels and chemicals is widely acknowledged. Over the past two decades, researchers around the globe have focused on the efficient enzymatic hydrolysis of lignocellulosic feedstocks and the fermentation of the resulting sugars into bulk and fine chemicals (1). Among the promising options for cost-effective lignocellulose degradation stands the application of cellulosomes, multi-enzyme complexes that efficiently orchestrate the degradation of (hemi)cellulose and thus play a significant role in nature’s carbon turnover (2). The cellulosome architecture allows to bring multiple (hemi)cellulases in close proximity to each other and to the substrate, resulting in superior degradation efficiencies compared to non-complexed enzymes. The high efficiency of these streamlined cellulosomes sparked the concept of engineering designer cellulosomes (DCs) with a tunable organization and composition. These DCs are built up by a chimeric cohesin-containing scaffoldin, which functions as a backbone to incorporate multiple chimeric docking enzymes (DEs). The species-specific interaction between the cohesin-dockerin modules ensures precise incorporation of multiple catalytic activities (3). DCs have often been proposed as an elegant alternative or complement to the expensive enzyme cocktails for lignocellulose breakdown (4). However, despite major advances in the field, DCs have not achieved their anticipated impact, with none being deployed for large-scale biomass conversion. While the DNA assembly of DC components (scaffoldin and DEs) was leveraged to the combinatorial level, thus eliminating an important technical hurdle, DC production can still not match this improved throughput at the protein level (5, 6). The cumbersome process of recombinant expression and purification of all DC components is at least one contributing reason why DCs have not yet found their way to the biorefinery (7).

To boost DC engineering toward an increased throughput level, their production by yeast cells is of great interest (8–11). The yeast surface display (YSD) approach is envisioned to achieve less time-, labor-, and resource-consuming DC production, as the spontaneous assembly of DCs on the yeast cell surface allows researchers to bypass many intermediate steps (7). Moreover, yeast surface-displayed DCs may also gain biological advantages not met by their soluble state, for example, higher thermostability and lower accessibility for proteases (8, 12). Finally, this approach enables DCs to take part in a consolidated bioprocess (CBP), as the displayed DCs degrade the available substrate, followed by uptake of the released monomeric sugars by the yeast cell and their use for fermentation. CBP has emerged as a promising technology to reduce the cost of the conventional biomass-to-ethanol process (10). Of relevance, DCs have been assembled on the yeast cell surface before to form a CBP. Even though *S. cerevisiae* has various suitable characteristics (e.g., high ethanol productivity, high inherent ethanol tolerance, resistance toward various inhibitors present in lignocellulosic biomass, and a generally regarded as safe status), the difficulty of producing multiple heterologous (hemi)cellulases in sufficient quantities is considered a major hurdle in its development as a CBP-host (10, 13–15). Indeed, it has been shown that the insufficient production of one or more DC components may lead to aberrant subpopulations (8, 10, 16). A detailed examination of *S. cerevisiae* to map the main bottlenecks of DC production and display remains unaddressed in the current body of literature.

The production of heterologous proteins in *S. cerevisiae* is known to face several bottlenecks including tightly controlled yeast quality control mechanisms, metabolic burden, and cellular stress responses (13, 17–20). Given these challenges and the difficulties associated with displaying balanced DCs on the yeast cell surface, gaining a comprehensive understanding of *S. cerevisiae*’s capacity as a DC expression host is crucial to enable an effective CBP. To address this unaddressed need, we here introduce the use of fluorescent protein complexes (FPCs), which mimic the DC architecture while facilitating convenient and precise detection of the different components on the yeast cell surface. By subjecting FPC-displaying yeast cells to population-wide flow cytometric and single-cell confocal microscopic analysis, our study offers a visual and quantitative tool to pinpoint these DC bottlenecks at a single-cell level. While flow cytometry (FC) allowed us to screen a high number of cells for their individual protein production levels, complementary confocal fluorescence microscopy enabled detailed FPC-displaying yeast cell imaging. This single-cell visualization revealed surprizing patterns in scaffoldin surface localization and the impact of metabolic burden, providing a crucial realistic view that directly informs why current single-host CBP systems often struggle with balanced, high-level expression of DC components.

## RESULTS AND DISCUSSION

### Development of a FPC

The spontaneous assembly of DCs on the yeast cell surface requires (i) the display of a scaffoldin and (ii) the secretion of DEs. After secretion into the medium, the DEs are expected to dock on the nearest available scaffoldin, relying on the strong and highly specific cohesin-dockerin interaction (*K*_*D*_ = 10^−12^ to 10^−7^ M) (7). We call this type of YSD system “self-assembly.” To build a homogenous yeast population, scaffoldins and DEs should be adequately expressed. DE stoichiometry is particularly important to harmonize enzymatic activities, while a satisfactory amount of functional scaffoldin needs to be displayed to retain secreted DEs to the cell. Therefore, it is crucial to examine the presence of each individual DC component to thoroughly evaluate *S. cerevisiae* as a DC display host. In this work, this was achieved through the introduction of a FPC. These FPCs adopt the same architecture as DCs, containing a chimeric cohesin-containing scaffoldin on which multiple dockerin-fused proteins can be precisely incorporated using species-specific cohesin-dockerin pairs. However, unlike DCs that incorporate carbohydrate-active DEs, we here use a fluorescent protein instead, introducing the fluorescent docking protein (FDP). By combining the detection of the FDP with immunostaining of the scaffoldin, the production of multiple FPC components by a single yeast cell can be evaluated in a single assay. Even though FPCs do not serve the same function as DCs, which efficiently degrade (hemi)cellulose polymers, and though protein expression and secretion is protein-dependent and thus different for FDPs compared to DEs, the similar architectures of FPCs are expected to offer a valuable representation of what can be expected of *S. cerevisiae* as a DC display host (Fig. S1).

For both the population-wide and single-cell analysis of FPC self-assembly on the yeast cell surface, a monovalent FPC was constructed. This complex contains a scaffoldin and one FDP. The scaffoldin consists of a carbohydrate-binding module (CBM) from *Clostridium thermocellum* and three cohesin modules originating from *Acetivibrio cellulolyticus* (*Ac*), *Bacteroides cellulosolvens (Bc*), and *Archeoglobus fulgidus *(*Af*), respectively. To allow for immunostaining, the scaffoldin is provided with a C-terminal V5-epitope. Scaffoldin display on the yeast cell surface is controlled by an inducible GAL1 promotor and facilitated through fusion to the Aga2p subunit of *S. cerevisiae*’s a-agglutinin cell-surface receptor. The FDP consists of a green fluorescent protein (GFP) fused with a flexible glycine-rich linker and an *Ac*-dockerin module. Expression of this FDP is controlled by a constitutive promotor, and a secretion signal facilitates secretion of the protein into the medium.

### Population-wide analysis of FPC expression

**Fig 1:**
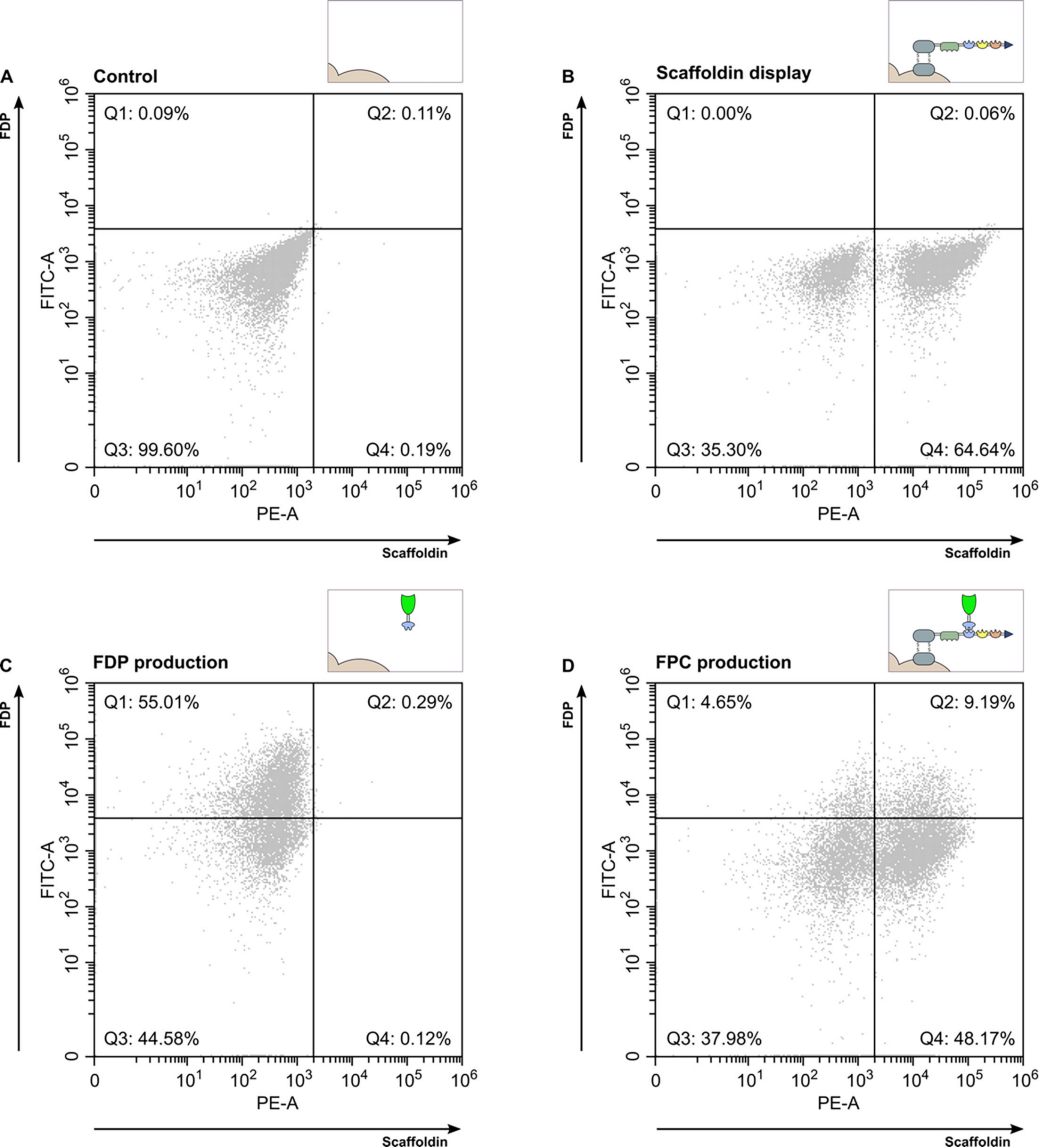
Population-wide analysis of FPC production. PE intensity is plotted on the *x*-axis and indicates scaffoldin display. Green fluorescent intensity, detected in the fluorescein isothiocyanate (FITC) channel, is plotted on the *y*-axis and indicates FDP production. Panel A depicts how the control strain was used to define quadrants. Panels B, C, and D show how the population of the scaffoldin-displaying, FDP-producing, and FPC-producing strain, respectively, is distributed over the different quadrants. The proportions of the population present in each quadrant are depicted in the plots. The inlets illustrate the analyzed yeast strains. Note that FDP production in panel C is only detected as a result of FDP accumulated in the secretory pathway, whereas in panel D, the FITC channel detects both intracellularly accumulated and cell surface-assembled FDP.

FC was used as a first approach to evaluate scaffoldin display and FDP production. Yeast populations expressing no, one, or both FPC component(s) were evaluated. As the FDP emits a green fluorescent signal once correctly folded, the cell’s FDP production can be linked to its fluorescence level. Detection of scaffoldin display was conducted through immunofluorescence labeling of the V5-epitope that is present at the C-terminus of each scaffoldin with a phycoerythrin (PE) fluorochrome-conjugated anti-V5 antibody. Additionally, the use of beads labeled with defined PE levels allowed us to determine the average number of displayed scaffoldins per cell, in the positively stained population. Figure 1 presents the FC data of the different yeast strains in dual-staining dot plots: PE intensity is plotted on the *x*-axis and represents scaffoldin display, whereas FDP intensity is plotted on the *y*-axis. This strategy allows us to map the portion of the population that performs display of the scaffoldin or production of the FDP. Also, the double-positive subpopulation that performs co-expression of both FPC components can be determined.

First, the distribution of the control strain (Fig. 1A) was used to draw gates defining the non-expressing (Q3), FDP-expressing (Q1), scaffoldin-expressing (Q4), and FDP and scaffoldin co-expressing (Q2) cells of the population. Second, Fig. 1B represents the population distribution of the scaffoldin-displaying strain. Scaffoldin display was detected on 64.70% (Q2 + Q4) of the cells of the population, with an average number of 13,756 anchored scaffoldin molecules per cell. This is consistent with other reported scaffoldin display systems. For example, Wen and colleagues determined approximately 18,000 copies of a trivalent scaffoldin anchored per cell (8). The observed heterogeneity, with a substantial fraction of cells not displaying scaffoldin, can be attributed to factors such as plasmid loss, challenges with correct folding or instability of the scaffoldin leading to undetectable V5 tags, and the inherent stochasticity of recombinant protein expression at the single-cell level. For the FDP-producing strain (Fig. 1C), 55.30% (Q1 + Q2) of the population exhibited a green fluorescent signal; however, in contrast to the scaffoldin-displaying population (separated Q3 and Q4), no distinct positive versus negative subpopulations were obtained. Notably, FDP-secreting cells were indirectly detected due to the accumulation of FDP in the secretory pathway in the cells. Finally, the last strain is tasked to simultaneously express both FPC components, and therefore theoretically assembles the full FPC (Fig. 1D). This strain exhibits a scaffoldin-displaying (Q2 + Q4), FDP-producing (Q1 + Q2), and co-expressing (Q2) subpopulation of 57.36%, 13.84% and 9.19%, respectively. Notably, Q4 also shows that 48.17% of the population is scaffoldin-positive but FDP-negative despite having the capacity for co-expressing both components. However, the continuous nature of FDP expression, without distinctly separated positive and negative populations as seen in Fig. 1C, can result in an underestimation of FDP-producing cells during gating.

An average number of 5,291 displayed scaffoldin molecules per cell was determined (compared to 13,756 anchored scaffoldin molecules per cell without FDP co-expressing). When comparing the FPC-producing strain with the scaffoldin-displaying strain, only a slight reduction (i.e., 57.36% vs 64.70%) in the percentage of cells that successfully display the scaffoldin was observed. These results suggest that while the scaffoldin-displaying capacity of yeast cells is not lost upon the production of the FDP, FDP production negatively impacts the amount of surface-displayed scaffoldins per cell. Indeed, the number of scaffoldin molecules per cell dropped by 61.54% when the yeast cells were tasked with the additional production of an FDP. Conversely, downscaled FDP expression can be observed when comparing the FDP- and FPC-producing yeast strains (55.30% vs 13.84%). Here, the amount of FDP-producing yeast cells decreased by 74.97% when tasked with additional scaffoldin display. Possibly, protein production levels decrease strongly because of an increased metabolic burden on the cell and/or saturation of the secretory pathway. Indeed, yeast cells able to self-assemble FPCs are expected to be exposed to a higher metabolic load resulting from increased heterologous protein expression. This metabolic burden is suggested to stem from the energy and metabolite costs of plasmid replication and recombinant protein production. As such, it is possible that this resource drain diverts the cellular machinery away from growth (FPC-producing yeast cultures showed ~4-fold and ~7.5-fold slower growth rates than scaffoldin- and FDP-producing yeast cultures, respectively) but also from the production of the FPC components. Overall, the producing cells may make a cellular trade-off between scaffoldin display and FDP production. Eventually, the combined expression of both the scaffoldin and FDP could still be detected in 9.19% of the yeast cells in the FPC-producing population.

### Single-cell analysis of FPC expression

The population-wide FC analysis revealed that co-expression of multiple FPC components is the major hurdle of the self-assembly system. Indeed, other reported DC self-assembly systems face the same problem of imbalanced expression levels when multiple components are simultaneously produced. Recombinant plasmid instability, metabolic burden, and saturation of the cellular secretion system have been suggested as possible causes (8, 10, 16, 21). An alternative approach for DC assembly on the yeast surface is the formation of a yeast consortium. In a consortium, multiple strains each expressing a dedicated DC component are combined to construct DCs via intercellular complementation. By dividing the stress and resource drain of maintaining and expressing foreign genes over multiple cells, higher expression levels can be achieved, although the ability to spontaneously assemble and display DCs is preserved (11, 12, 22–24). Therefore, this multiple host cell system is an appealing option to explore for the optimization of FPC component co-expression.

**Fig 2:**
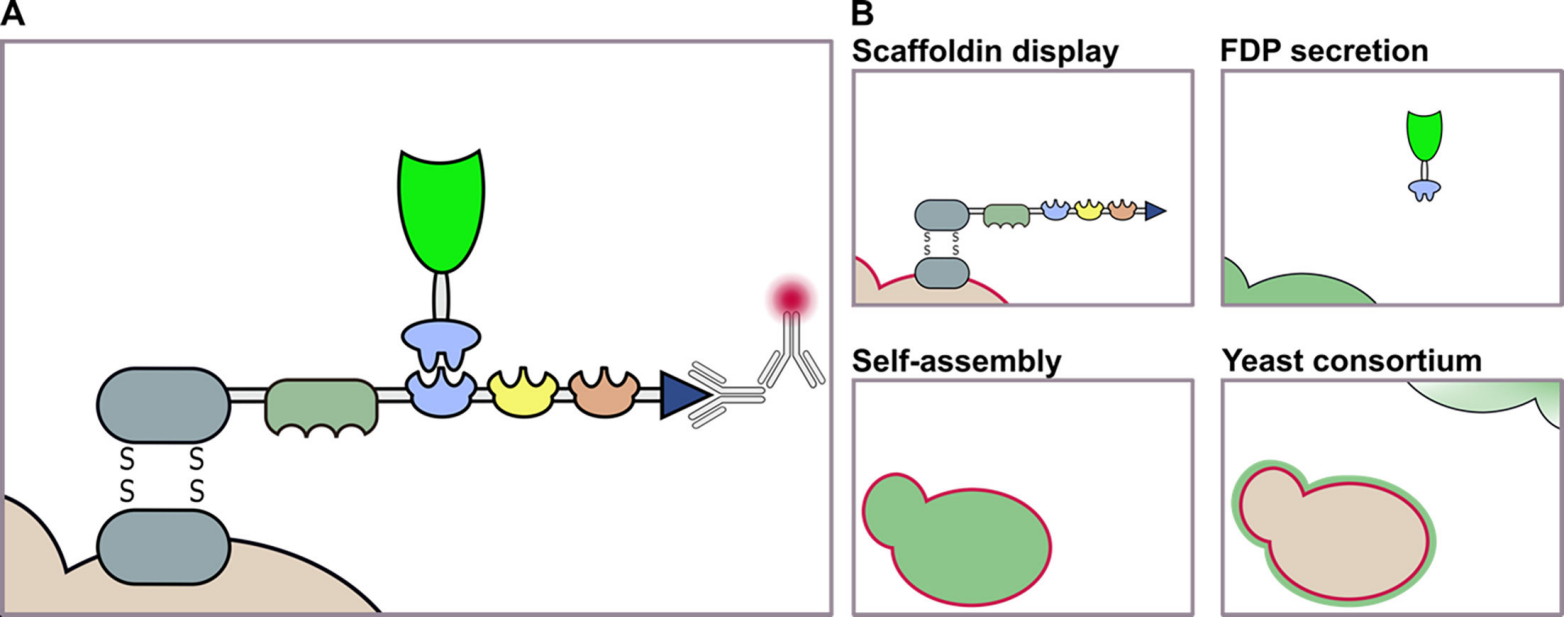
Visualization of yeast cells displaying the FPC. (A) Both FPC components can be individually detected. Produced FDPs emit a green fluorescent signal. The displayed scaffoldin can be immuno-stained using an anti-V5 primary antibody and a pink fluorochrome-conjugated secondary antibody. (B) Expected outcomes of visualized yeast cells. Upper left: scaffoldin-displaying yeast cells are expected to show a pink-stained surface. Upper right: yeast cells producing FDPs are expected to exhibit green fluorescence. Lower left: the self-assembly system tasks yeast cells to perform scaffoldin display and FDP expression. Consequently, the cells are expected to show intracellular green fluorescence and a pink and green surface. Lower right: a synthetic yeast consortium is formed by combining scaffoldin-displaying yeast cells with the supernatant of FDP-secreting yeast cells (blurred green cell). The assembly of secreted FDPs on surface-displayed scaffoldins is presumed to manifest as both pink and green fluorescent labeling of the yeast cells' surface.

FPC-displaying yeast cells by self-assembly and through a yeast consortium were examined on the single cell level, using confocal fluorescence microscopy. This visualization sheds light on how cells of the different display systems deal with the challenging co-expression of multiple FPC components. FPC self-assembly was achieved by tasking yeast cells with both scaffoldin display and FDP secretion, as described before (Fig. 1D). A synthetic yeast consortium was formed by separately culturing yeast strains performing scaffoldin display or FDP secretion. Consequently, the supernatant of the FDP-producing strain was combined with scaffoldin-displaying yeast cells to allow the surface assembly of FPCs. As before, the different FPC components can be individually examined. The FDP emits green fluorescence once correctly folded in the cell, and displayed scaffoldins can be imaged upon immunostaining. For this, an anti-V5 primary antibody and a secondary antibody conjugated with a pink fluorochrome (Alexa Fluor Plus 647) were used. The possible outcomes of immunofluorescence microscopy of yeast cells assembling this FPC are depicted in Fig. 2. FPC self-assembly is expected to manifest as green fluorescent cells with a pink fluorescent surface. Contrarily, a yeast consortium combines extracellular FDPs with scaffoldin-displaying cells. If the FDPs are successfully secreted into the medium and able to dock on the displayed scaffoldins, these FPC-displaying yeast cells should exhibit both green and pink fluorescence on their surface.

Prior to the analysis of the different strains, several controls were examined. A negative (secondary antibody only staining) and scaffoldin non-induced control did not show any fluorescent signal. These results prove the absence of non-specific binding of the secondary antibody and the strong catabolite repression of the GAL1 promotor in glucose-containing medium (25). In addition, also a positive control was examined. This control entailed stained scaffoldin-displaying yeast cells, whose surfaces showed to be brightly fluorescent. All controls are presented in Fig. S2.

#### Construction of self‐assembled FPCs on the yeast surface

**Fig 3:**
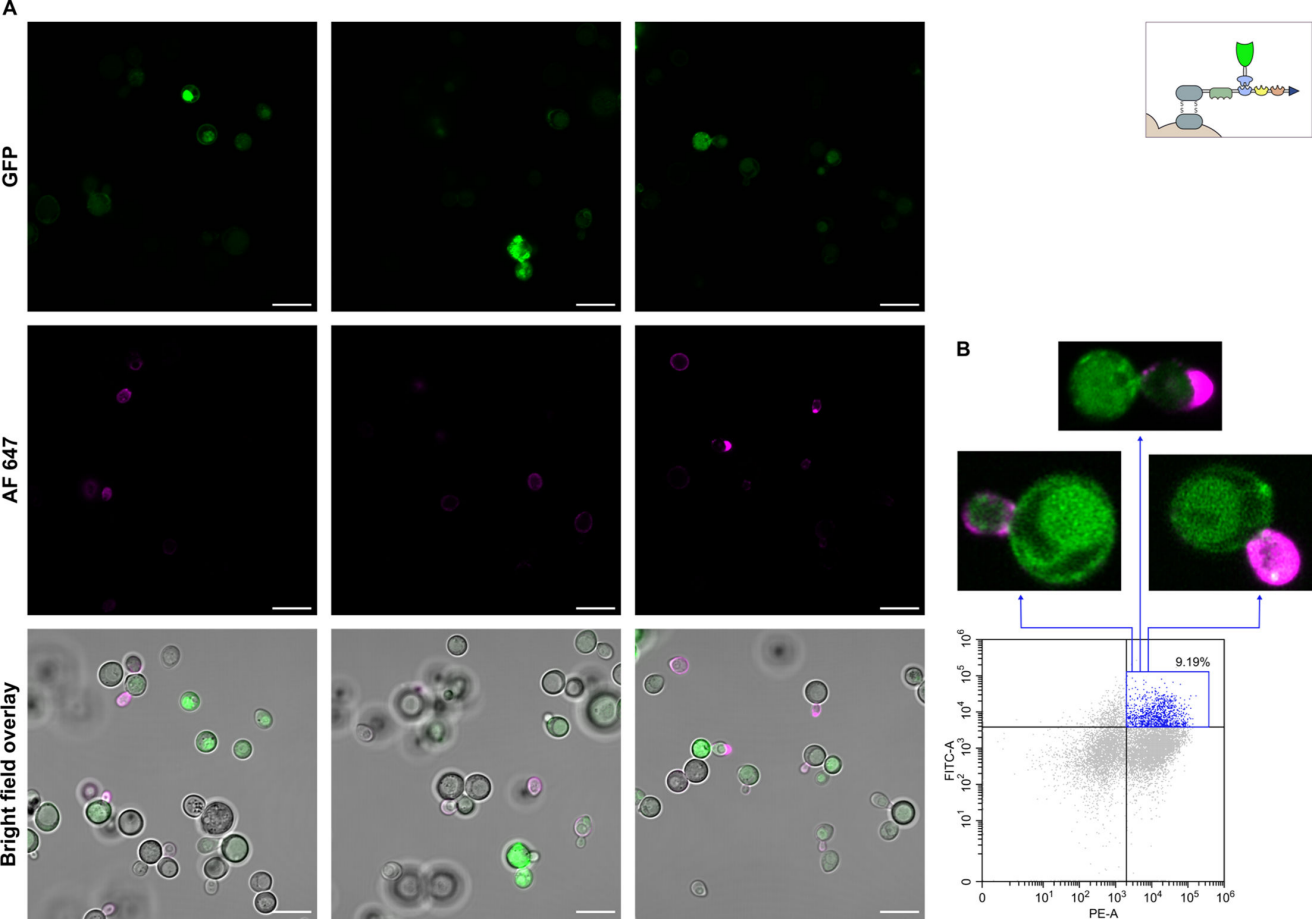
Microscopic analysis of FPC-producing yeast cells, employing the self-assembly system. (A) Confocal images of yeast cells performing self-assembly of the FPC (depicted in upper right corner). Cells producing FDPs show green fluorescence (GFP). Cells performing scaffoldin display exhibit pink fluorescence on their surface (AF 647). In the bottom row, an overlay of the fluorescence and bright field channels image is depicted. Scale bars correspond to 10 µm. (B) The cellular analysis can be coupled to the previously reported FC data, analyzing a population of 10,000 cell events. For this strain, a co-expressing population of 9.19% was demonstrated. The cropped pictures show that the double-positive cells imaged under the microscope mostly occur as an FDP-expressing mother cell with a scaffoldin-displaying bud. Fluorescence intensity in these cropped pictures was raised to obtain optimal visualization. A 3D visualization of a budding yeast cell performing FPC self-assembly can be found in Movie S1.

FPC display in terms of self-assembly was subjected to an in-depth evaluation. About 100 cells from different microscopic fields were imaged under a confocal microscope and analyzed for green (GFP) and pink (AF 647) fluorescence (Fig. 3A; Table S1). This visual examination provided qualitative insights into the cellular distribution and co-localization of FPC components at the single-cell level. First, a substantial number of cells exhibit clear green fluorescence as a result of FDP production. The strong intracellular fluorescent signal observed in multiple cells might indicate that the FDP is to some extent retained in the secretory pathway. However, we are unable to pinpoint the exact rate-limiting step(s) in FDP expression, which could be at the level of the endoplasmic reticulum, Golgi complex, or vesicle transport. A co-staining with markers to visualize the different intracellular elements may provide a more detailed view (26, 27). FDP expression was visually observed in approximately 73% of the analyzed cells, providing qualitative insight into the distribution of FDP-producing cells. This is a notably higher percentage than the FDP-producing population reported by FC analysis (13.84%, Fig. 1D Q1 + Q2). This could be partly attributed to the absence of a distinct positive and negative population in the FC data (Fig. 1C), resulting in an underestimation of FDP-producing cells when gating the positive population. Second, the number of cells that perform scaffoldin display accounted for 25%, which is a considerably lower amount than the 57.36% reported by FC (Fig. 1D). We note that the percentages reported here are based on a visual evaluation and as such, do not reflect differences in intensity. Possibly, FC has a lower detection limit and may as such identify a larger portion of the population as positive. In either case, the intercomparison between FC and microscopy data must be considered with caution since these different techniques handle a distinct range of cell counts, and the confocal analysis here is primarily qualitative and observational based on 100 cells from random microscopic fields, providing detailed single-cell views rather than statistically robust quantification of cell populations. Remarkably, scaffoldin display was predominantly detected in the buds of reproducing yeast cells. In the scaffoldin-displaying subpopulation, 84% of the cells show a positively stained bud. Moreover, in 76% of the cases, display is completely shifted to the bud and absent in the mother cell. Consequently, the majority of the scaffoldin and FDP co-expressing cells (amounting to 9.19% as shown by FC, Fig. 1D) emerge as an FDP-expressing mother cell with a scaffoldin-displaying bud (Fig. 3B).

The control non-induced scaffoldin condition showed intracellular FDP production but no scaffoldin display (Fig. S3). A 3D visualization of a budding yeast cell performing FPC self-assembly can be found in Movie S1.

#### FPC display facilitated by a synthetic yeast consortium

**Fig 4:**
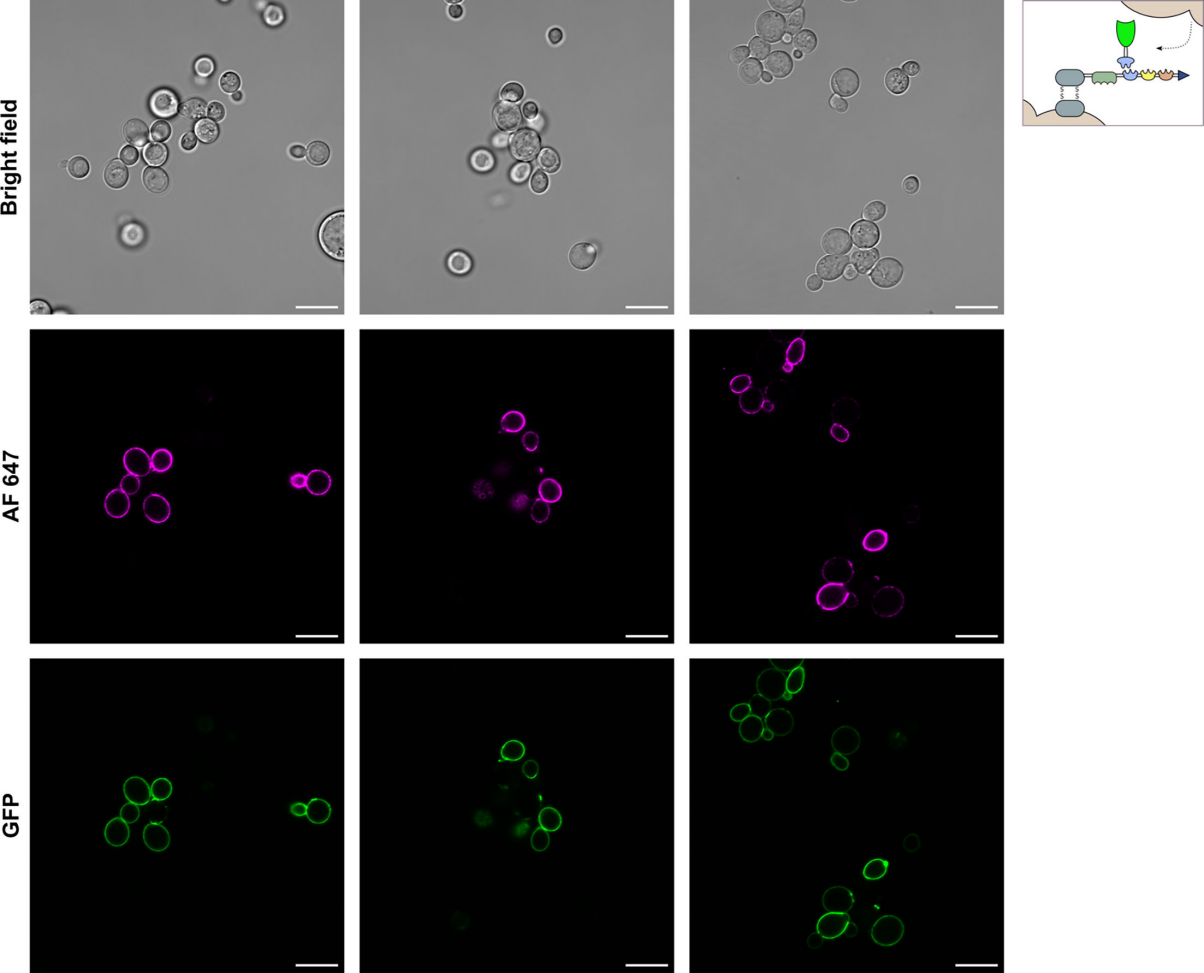
Microscopic analysis of FPC YSD by the formation of a synthetic yeast consortium. Confocal images of yeast cells displaying the FPC by intercellular complementation (depicted in upper right corner). Cells performing scaffoldin display exhibit pink fluorescence on their surface (AF 647). FDPs, delivered by the supernatant of a FDP-secreting strain, are able to dock on the displayed scaffoldins and exhibit a green fluorescent signal on the cell’s surface (GFP). Scale bars correspond to 10 µm. A 3D visualization of two budding yeast cells of the synthetic yeast consortium can be found in Movie S2.

Next to FPC display achieved by the self-assembly system, FPCs were assembled via intercellular complementation in a synthetic yeast consortium. Approximately 100 cells of the consortium containing the scaffoldin and externally added FDP (taken from different microscopic fields) were analyzed for their green (GFP) and pink (AF 647) fluorescent signal (Fig. 4; Table S2). This provided visual evidence of successful FPC assembly on the cell surface within the consortium approach. A visually quantified 56% of the imaged cells show a brightly pink fluorescent surface and thus exhibit successful scaffoldin display. In contrast to the self-assembling strain, the scaffoldin-displaying population here falls within the same range as the 64.70% reported by FC (Fig. 1B). The fact that more cells show visible scaffoldin display on the confocal images suggests a higher fluorescence intensity of the cells and therefore more scaffoldin molecules to be displayed on the surface when compared to the self-assembling strain. Indeed, this is consistent with the diminished cellular scaffoldin quantities resulting from the production of an additional FDP, as reported by FC analysis (Fig. 1B versus Fig. 1D). As such, the advantages of dividing the production of FPC components over multiple host cells in a yeast consortium can be clearly observed here. Interestingly, a considerably smaller percentage of the positively stained cells showed scaffoldin display on the buds. Compared to the single host system where scaffoldin display was completely shifted to the bud in 76% of the cases, this proportion only accounted for 12% here. Moreover, cells that perform scaffoldin display also show a bright green fluorescence on their surface. This clearly confirms the effective secretion of FDPs into the extracellular medium and shows that the high-affinity interactions between the scaffoldin and FDPs were sufficient for direct FPC assembly on the yeast cell surface.

The non-induced condition exhibited neither a pink nor green fluorescence signal at the cell wall, which confirms the absence of non-specific adsorption of FDPs to the yeast cells (Fig. S4). A 3D visualization of two budding yeast cells of the synthetic yeast consortium can be found in Movie S2.

#### The bud tip as cellular hot spot for scaffoldin display

**Fig 5:**
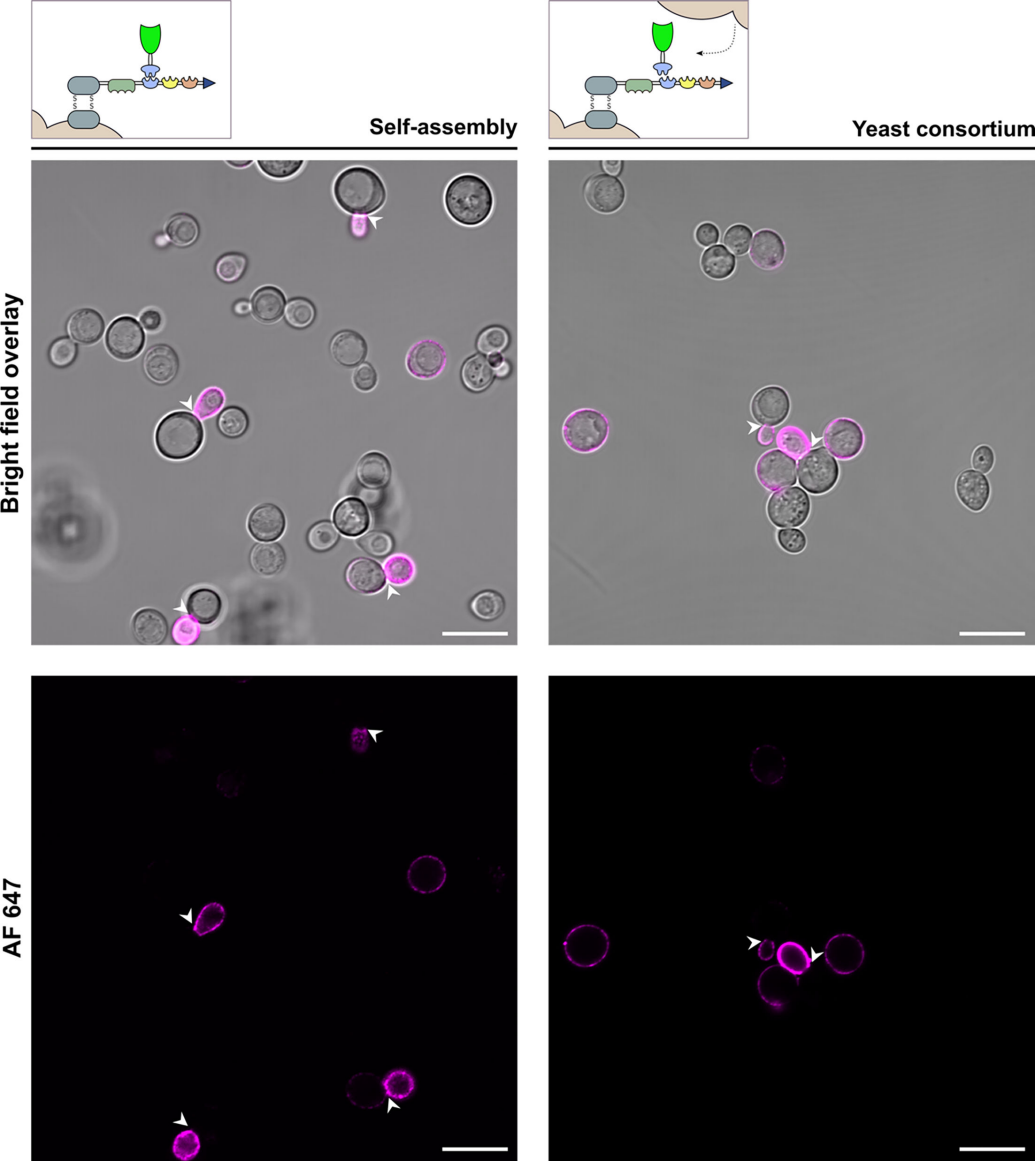
Scaffoldin localization on the surface of cells that perform self-assembly (left) and are part of a synthetic yeast consortium (right). Representative microscopic fields are depicted. Emerging buds are marked by a white arrowhead. Scale bars correspond to 10 µm. On the left confocal images, four scaffoldin-displaying buds can be detected. From these buds, only one mother cell shows a (rather weak) fluorescent signal as well. Also, one singular cell shows to perform display. On the confocal images presented on the right, two fluorescent-stained emerging buds are marked. Here, the mother cells do not show scaffoldin display either. However, four singular scaffoldin-displaying cells can be detected. This pattern of scaffoldin display also emerges on the confocal images presented in Fig. 3 and 4. We note that the fluorescence intensity of the images with self-assembling yeast cells (left) was raised for an adequate visualization. In reality, this fluorescent signal is ~5-fold less bright and as such significantly lower than signal emitted by the scaffoldin-displaying cells of a synthetic consortium.

As mentioned above, cells tasked with the self-assembly of FPCs mainly channel their scaffoldin displaying capacity to the newly emerging bud. This effect strongly manifests, as scaffoldin display is almost non-existent in mother and single cells. Cells in the consortium also exhibit scaffoldin-displaying buds. However, a substantial amount of mother and single cells displays the scaffoldin as well (Fig. 5).

In fact, the secretion of proteins at the tip of an emerging bud is a known phenomenon in *S. cerevisiae* (28). In the trans-Golgi network, proteins that are destined to be secreted (e.g., cell wall proteins) are sorted into secretory vesicles. These vesicles are transported along actin cables to exocytic sites located in the cell membrane at the bud tips, which are regions of active growth (29). Interestingly, research has shown that this is also the fact for the expression of heterologous proteins. Puxbaum and colleagues reported secretion of a recombinant protein fused to an agglutinin cell wall anchor through the bud. Secretion started at the bud tip, and as the bud grew, the protein was present over the entire bud surface, but not on the mother cell (26). Moreover, Yang et al. (30) showed that both recombinant protein secretion and surface display can be improved by upregulating genes involved in budding.

Taken together, we hypothesize that scaffoldin and FDP co-expression in FPC self-assembling yeast cells predominantly occurs as an FDP-producing cell with a scaffoldin-displaying bud (Fig. 3B). Bud formation is a cyclic process, and as such, a particular cell probably only performs scaffoldin display at certain time intervals. As almost no scaffoldin-displaying singular cells were imaged, it remains unclear what happens to the display capacity of the daughter cell once it defuses from the mother cell. One potential approach to explore this phenomenon further could be through time-lapse microscopy. In such an experiment, a cell division event can be monitored over time (26). In either case, this phenomenon throws an additional realistic view on the heterogeneity of FPC display and its impact on the concept of displaying DCs to form a CBP, as this discontinuous display of the scaffoldin will disrupt DE immobilization and colocalization.

Yeast cells that are exclusively tasked with scaffoldin display show this phenomenon to a much lesser extent. It appears that when cells are relieved of FDP expression, and as such experience less stress, the scaffoldin can also be displayed on the mother cell surface and is not lost after bud diffusion. It is known that mother cells have a selfless nature and retain, for example, damaged endoplasmic reticulum and factors that contribute to cell aging, presumably to increase the lifespan of the new daughter cells (27, 31). As such, it is conceivable that the buds are more “fit” to manage scaffoldin-expression compared to the mother cell, and this effect becomes most apparent under high metabolic burden.

To conclude, our results suggest that the metabolic load of producing multiple FPC components may manifest as a reduced surface area available for scaffoldin display. The discontinuous and localized display of the scaffoldin within a single cell presents a significant practical challenge for achieving consistent and efficient enzyme immobilization and colocalization necessary for optimal lignocellulose saccharification. In contrast, the more widespread and homogeneous display observed within a consortium setup offers a distinct advantage, circumventing these single-host limitations in a CBP setup.

### Conclusions

In this work, *S. cerevisiae* was thoroughly evaluated for its application potential as a DC display host. For this, we report the construction and use of an FPC. Each component of this FPC is fluorescent or can be fluorescently labeled, allowing convenient detection. Moreover, the FPC mimics the DC architecture and can therefore be used to assess the yeast cells’ effectiveness in displaying DCs. Our approach based on the highly complementary combination of FC and confocal microscopy gave a realistic and detailed view on the single cell level regarding FPC assembly on the yeast cell surface. While FC provided robust population-level quantitative data, the confocal microscopy offered in-depth qualitative insights into the heterogeneity and localization patterns of FPC components. Although each component of the FPC could be expressed by *S. cerevisiae*, FDP expression showed to have a major impact on scaffoldin display efficiencies. This effect manifested as lower display levels and discontinuous scaffoldin display throughout the cell cycle. The finite capacity of yeast cells to produce heterologous protein and their proneness to the effects of metabolic burden are suggested as major bottlenecks for efficient DC self-assembly by *S. cerevisiae*. Our results demonstrate the practical hurdles of this single host cell system to restrict the efficiency of FPC display. Instead, the distribution of FPC component production over multiple host cells in a yeast consortium allows more consistent production and assembly of FPCs on the yeast cell surface. In conclusion, these findings provide us with greater insights into the yeast cell’s ability to assemble protein complexes on its surface, providing guidance for the future design and implementation of yeast-displayed DCs in CBP systems. The in-depth investigation exposed new limitations of DC display that are till today ignored in the current body of literature.

## MATERIALS AND METHODS

### Plasmid construction

All DNA constructs were assembled using the VersaTile technique, a DNA assembly method dedicated to the simple construction of multi-modular proteins (5, 32). First, the coding sequence of a scaffoldin comprizing one CBM and three cohesin domains was constructed and ligated into the scaffoldin-display vector pVTD20. To obtain the pVTD20 vector, the commercially available pYD1 yeast display vector (Addgene (33) was made compatible with the VersaTile technique (Fig. S5). During a VersaTile assembly reaction, the scaffoldin coding sequence is assembled in the pVTD20 vector as a 3′ fusion to the Aga2p DNA sequence, forming an Aga2p-scaffoldin fusion construct. The used yeast strain has the Aga1p gene stably integrated into its genome, and the expression of both Aga1p and Aga2p is regulated by the galactose inducible GAL1 promotor. As such, when culturing the cells in a medium supplemented with galactose, the production of Aga1p and Aga2p-scaffoldin fusion construct is induced, whereafter the synthesized proteins associate within the secretion pathway and are exported to the cell surface (34). Second, the coding sequence of a FDP consisting of a GFP, flexible linker, and dockerin domain was constructed and ligated into the FDP-secretion vector pVTD35 that is equipped with an α-factor signal peptide for secretion. The pVTD35 was newly developed and ordered through Azenta Life Science, Germany (Fig. S6). The FDP coding sequence is preceded by a constitutive promotor, selected from the semi-synthetic yeast promotor library created by Decoene et al. (35), and the α-factor signal peptide DNA sequence (36). Downstream of the FDP coding sequence, the short synthetic terminator T_synth2_ selected from the *S. cerevisiae* terminator set constructed by Curran et al. (37), is present. Annotated sequences of both DNA constructs can be found in Table S3. Constructs were confirmed by Sanger sequencing.

### Strains and growth media

*E. coli* TOP10 cells were used for cloning procedures and plasmid storage. These strains (Agilent Technologies) were routinely grown at 37°C and 180 rpm in lysogeny broth (LB) medium (1% [wt/vol] tryptone, 0.5% [wt/vol] yeast extract, and 1% [wt/vol] NaCl) or on LB plates supplemented with 1.5% (wt/vol) of agar. For proper selection of the *E. coli* clones, LB was supplemented with 50 µg/mL kanamycin, 25 µg/mL zeocin and/or 5% (wt/vol) sucrose. When zeocin was required for selection, low-salt LB medium (1% (wt/vol) tryptone, 0.5% [wt/vol] yeast extract, and 0.5% [wt/vol] NaCl) was used.

*S. cerevisiae* strain EBY100 (*MAT*a *AGA1::GAL1-AGA1::URA3 ura3-52 trp1 leu2*Δ*200 his3*Δ*200 pep4::HIS3 prbd1.6 R can1 GAL*) was acquired from ATCC (cat. no. MYA-4941) and used as expression host. Untransformed yeast strains were grown in yeast peptone dextrose (YPD) medium (1% (wt/vol) yeast extract, 2% (wt/vol) peptone, 2% (wt/vol) glucose). *S. cerevisiae* EBY100 transformants containing the pVTD20 vector were grown in synthetic dextrose casamino acid (SDCAA) medium (2% [wt/vol] glucose, 0.67% [wt/vol] yeast nitrogen base, 0.5% [wt/vol] casamino acids, 38 mM Na_2_HPO_4_, and 62 mM NaH_2_PO_4_) and induced in synthetic galactose casamino acid (SGCAA) medium (2% [wt/vol] galactose, 0.67% [wt/vol] yeast nitrogen base, 0.5% [wt/vol] casamino acids, 38 mM Na_2_HPO_4_, and 62 mM NaH_2_PO_4_). *S. cerevisiae* EBY100 transformants containing the pVTD35 vector were grown in YPD medium supplemented with 100 µg/mL zeocin. Yeast strains containing both vectors were grown and induced in SDCAA and SGCAA media, respectively, supplemented with 100 µg/mL zeocin. All strains were grown at 30°C, and liquid cultures were shaken at 220 rpm. To solidify media, 1.5% (wt/v) agar was added.

### Yeast co-transformation and verification

#### Yeast (co-)transformation

Production of electrocompetent yeast cells and yeast transformations were carried out as described in the electroporation method of Cregg et al. (38). Additionally, 2 mL of YPD was added to the cells immediately after electroporation. This transformation mix was then incubated at 30°C and 220 rpm for 1 hour to allow cell recovery. Afterward, cells were grown in the appropriate selective medium. To obtain yeast cells with the verified pVTD20 vector, pVTD35 vector, or both, different yeast transformation protocols were carried out. First, scaffoldin-displaying yeast cells were obtained by performing a transformation of the EBY100 strain with the pVTD20 vector. Afterward, strains were grown on selective SDCAA plates for 3–4 days at 30°C. Second, FDP-secreting yeast cells were obtained by transforming the pVTD35 vector in strain EBY100. After transformation, cells were washed with phosphate-buffered saline (PBS) (137 mM NaCl, 2.7 mM KCl, 10 mM Na_2_HPO_4_, and 1.8 mM KH_2_PO_4_; pH 7.4) and cultured in YPD medium supplemented with 100 µg/mL zeocin for 2 days at 30°C and 220 rpm. Subsequently, cells were washed with PBS, plated on YPD plates supplemented with 100 µg/mL zeocin, and incubated at 30°C for 3–4 days. Finally, yeast cells can be provided with both vectors. For this, electrocompetent yeast cells were produced starting from the pVTD20-containing EBY100 strain. Subsequently, a transformation of these cells was performed with the pVTD35 vector. After transformation, cells were washed with PBS and cultured in SDCAA medium supplemented with 100 µg/mL zeocin for 2 days at 30°C and 220 rpm. After washing with PBS, the cells were selected on SDCAA plates supplemented with 300 µg/mL zeocin for 3–4 days, at 30°C.

#### Verification of plasmid uptake

Correct *S. cerevisiae* strains were confirmed by performing PCR. Per strain, several colonies were picked and grown in the appropriate selective medium. After this, cells were harvested and incubated with 6 U of zymolyase for 1 hour at 37°C. Plasmid DNA was extracted using the GeneJET Plasmid Miniprep Kit (Thermo Fisher Scientific), following the manufacturer’s guidelines. Subsequently, inserts of the extracted pVTD20 and pVTD35 vectors were amplified with Phusion DNA polymerase (1 U) (Thermo Fisher Scientific), following the manufacturer’s instructions. An overview of the primers used for verification is given in Table S4. The sizes of the amplified DNA fragments were confirmed through gel electrophoresis.

### Flow cytometric analysis of surface display

All antibodies were obtained from ThermoFisher Scientific. A Cytoflex flow cytometer (Beckman Coulter, UGent CORE Flow Cytometry) was used. Data were analyzed using the Cytexpert software (Beckman Coulter). Yeast strains were cultured in 24-well plates with round well bottoms. The negative control strain carried the pVTD20 vector with no insert. Strains provided with the pVTD20 vector (control strain and scaffoldin-displaying strain) were grown in SDCAA medium. Strains containing both the pVTD20 and pVTD35 vector (FDP- and FPC-producing strains) were grown in SDCAA medium supplemented with 100 µg/mL zeocin. The 24-well plates were placed on a plate shaker at 700 rpm for 18 hours at 30°C. Subsequently, cells were harvested by centrifugation (5 minutes, 2,000 *g*), washed with PBS, and diluted to 1 OD_600_/mL in fresh SDCAA or SGCAA medium, supplemented with 100 µg/mL zeocin where applicable. Cultures were incubated for 20 hours at 30°C and 700 rpm. Thereafter, about 10^6^ cells were washed twice with PBSA (PBS supplemented with 0.5% (wt/vol) bovine serum albumin (BSA) and 2 mM EDTA, pH 7.4) and resuspended in 100 µL antibody solution, containing a 12.5-fold dilution of PE-conjugated V5-tag monoclonal antibody (12-6796-42) in PBSA. Non-stained samples were resuspended in 100 µL PBSA instead. Cells were incubated for 30 minutes at 4°C followed by two wash steps with PBSA. Flow cytometric analysis was performed on single biological samples, with data collected from 10,000 events per sample, representing a robust technical replicate for within-sample variability. Additional controls were used to compensate for spectral bleed-through. Quantification of scaffoldin surface display was performed using the Quantibrite PE fluorescence quantitation kit (BD Biosciences) according to the manufacturer’s protocol. Briefly, the Quantibrite beads were used to establish a standard curve of PE fluorescence intensity versus the number of PE molecules. This allowed for the conversion of the mean fluorescence intensity of the scaffoldin-displaying cells to the number of PE molecules bound per cell, which directly correlates with the amount of scaffoldin displayed on the cell surface.

### Confocal laser scanning microscopic imaging of surface display

All antibodies were obtained from ThermoFisher Scientific. As before, different types of yeast strains were cultured in different selective media, and the negative control strain carried the pVTD20 vector with no insert. First, the scaffoldin-expressing strain, containing the pVTD20 vector, and the FPC-producing strain, containing both the pVTD20 and pVTD35 vector, were grown and induced as described above. Second, yeast strains that perform FDP expression were cultured in YPD medium supplemented with 100 µg/mL zeocin and 10 mM CaCl_2_. The cells were grown in 24-well plates, which were placed on a plate shaker at 700 rpm for 40 hours at 30°C. To form the synthetic consortium, culture supernatant of the FDP-expressing strains was collected and concentrated threefold using a Vivaspin 20 centrifugal concentrator (10 kDa cutoff, Sigma-Aldrich). About 2 × 10^6^ scaffoldin-displaying cells were harvested and resuspended in 1 mL concentrated supernatant. To allow FPC formation, 10 mM CaCl_2_ was added to the cell mixture, which was then incubated for 1 hour at 37°C without shaking.

Both the yeast cells of the synthetic consortium and yeast cells performing self-assembly were collected and washed twice with PBSA. A cell amount of 2 × 10^6^ was resuspended in 200 µL primary antibody staining solution (200-fold dilution of V5-tag monoclonal rabbit antibody [SY30-01] in PBSA) and incubated for 30 minutes at 4°C. No primary antibody was added to the negative control. This first staining step was followed by two wash steps with PBSA. Consequently, the cells were resuspended in 200 µL secondary antibody solution (400-fold dilution of Alexa Fluor plus 647 conjugated goat anti-rabbit antibody [A32733] in PBSA). After a last incubation step of 30 minutes at 4°C, the cells were washed twice and finally resuspended in 20 µL PBSA. As such, a final cell concentration of 10^8^ cells per mL was obtained. Finally, 2 µL cell suspension was pipetted on a slide and observed under the Zeiss LSM880 Fast AiryScan microscope, using consistent settings. Images were processed with the ZEN black edition system 2.3 (Zeiss).

## SUPPLEMENTAL MATERIAL

Supplemental tables and figures spectrum.00750-25-s0001.pdf Tables S1 to S4 and Fig. S1 to S6.

Supplemental movies legend spectrum.00750-25-s0002.docx Legends for Movies S1 and S2.

Movie S1 spectrum.00750-25-s0003.mp4 3D visualization of a budding yeast cell performing FPC self-assembly. This visualization shows that FDP production (green) and scaffoldin display (prink) are divided over the mother and daughter cell, respectively.

Movie S2 spectrum.00750-25-s0004.mp4 3D visualization of two budding yeast cells of the synthetic yeast consortium. FPC display is detected on the surface of the cells, as both pink and green fluorescent signal dedicated to the presence of the scaffoldin and FDP, respectively, can be observed. Note that the fluorescent signal is the strongest at the surface of newly emerging buds.

ASM does not own the copyrights to Supplemental Material that may be linked to, or accessed through, an article. The authors have granted ASM a non-exclusive, world-wide license to publish the Supplemental Material files. Please contact the corresponding author directly for reuse.
