# Supplementary material for: Single-cell analysis of yeast surface display for designer cellulosome applications using a fluorescent protein complex
Source: Microbiol Spectr. 2025 Aug 12;13(9):e00750-25. doi: 10.1128/spectrum.00750-25 (PMC12403707; doi:10.1128/spectrum.00750-25)
Supplement: Supplemental tables and figures — Tables S1 to S4 and Fig. S1 to S6. [file spectrum.00750-25-s0001.pdf]

Table S1. Detailed cell count of the different phenotypes observed in the self-assembly approach. Cells from seven random microscopic fields were evaluated and assigned to one of the five different phenotypes. Percentages representing the respective proportions compared to the total cell count and to the total number of scaffoldin-displaying cells (where appropriate) are shown.

								Total cell count	% of total	% of scaffoldin-displaying cells
**PICTURE**	**1**	**2**	**3**	**4**	**5**	**6**	**7**			
**Number of cells**	10	20	17	23	6	12	14	102	100	
**Number of scaffoldin-displaying cells**	2	4	1	5	1	4	8	25	25	100
**Number of scaffoldin-displaying cells with stained buds**	2	4	1	4	1	2	7	21	21	84
**Number of FDP-producing cells**	7	13	10	17	3	10	14	74	73	
**Number of scaffoldin-displaying and FDP-producing cells**	2	3	1	5	1	4	8	24	24	
**Number of FDP-producing cells with scaffoldin-displaying bud**	2	3	1	4	1	2	6	19	19	76

Table S2. Detailed cell count of the different phenotypes observed in the consortium approach. Cells from seven random microscopic fields were evaluated and assigned to one of the five different phenotypes. Percentages representing the respective proportions compared to the total cell count and to the total number of scaffoldin-displaying cells (where appropriate) are shown.

								Total cell count	% of total	% of scaffolding-displaying cells
**PICTURE**	**1**	**2**	**3**	**4**	**5**	**6**	**7**			
**Number of cells**	13	8	8	12	6	12	18	77	100	
**Number of scaffoldin-displaying cells**	7	4	4	6	3	6	13	43	56	100
**Number of scaffoldin-displaying cells with stained buds**	1	0	3	2	1	3	3	13	17	30
**Number of scaffoldin-displaying cells with only stained buds**	0	0	1	2	0	2	0	5	6	12
**Number of FDP-displaying cells**	7	4	6	6	3	9	13	48	62	
**Number of scaffoldin- and FDP-displaying cells**	7	4	4	6	3	6	12	42	55	

Table S3: DNA constructs assembled with the VersaTile technique (1, 2). For both the scaffoldin and FDP construct, the incorporated modules are presented in column two and the full DNA sequence (presented in the 5’-3’ direction) is given in column three. The coding sequences of the incorporated modules are highlighted in bold. The underlined sequences represent the short linkers between adjacent modules, inherent to the VersaTile technique. The italicized sequences are present on the vector backbone and can also entail modules. In particular, the pVTD20 vector for scaffoldin construction fuses the Aga2p module and V5 epitope coding sequences to the 5’ and 3’ termini of the constructed DNA sequence, respectively (see Figure S1). Columns four and five provide the length of the DNA sequence and molecular weight of the corresponding protein.

Construct	Modules	DNA sequence (5’-3’)	Sequence length (bp)	Molecular weight (Da)
Scaffoldin	Aga2p—<br>*Ct*-CBM3a—<br>Coh-*Ac*—<br>Coh-*Bc*—<br>Coh-*Af*—<br>V5	***ATGCAGTTACTTCGCTGTTTTTCAATATTTTCTGTTATTGCTTCAGTTTTAGCACAG***<br>***GAACTGACAACTATATGCGAGCAAATCCCCTCACCAACTTTAGAATCGACGCCGTAC***<br>***TCTTTGTCAACGACTACTATTTTGGCCAACGGGAAGGCAATGCAAGGAGTTTTTGAA***<br>***TATTACAAATCAGTAACGTTTGTCAGTAATTGCGGTTCTCACCCCTCAACAACTAGC***<br>***AAAGGCAGCCCCATAAACACACAGTATGTTTTT****AAGCTTCTGCAGGCTAGTGGTGGT*<br>*GGTGGTTCTGGTGGTGGTGGTTCTGGTGGTGGTGGTTCTGCTAGCATGACTGGTGGA*<br>*CAGCAAATGGGTCGGGATCTGTACGACGATGACGATAAGGTACCAGGATCCAGTGTG*<br>*GTGGAATTCACCATG***GTATCAGGCAATTTGAAGGTTGAATTCTACAACAGCAATCCT**<br>**TCAGATACTACTAACTCAATCAATCCTCAGTTCAAGGTTACTAATACCGGAAGCAGT**<br>**GCAATTGATTTGTCCAAACTCACATTGAGATATTATTATACAGTAGACGGACAGAAA**<br>**GATCAGACCTTCTGGTGTGACCATGCTGCAATAATCGGCAGTAACGGCAGCTACAAC**<br>**GGAATTACTTCAAATGTAAAAGGAACATTTGTAAAAATGAGTTCCTCAACAAATAAC**<br>**GCAGACACCTACCTTGAAATAAGCTTTACAGGCGGAACTCTTGAACCGGGTGCACAT**<br>**GTTCAGATACAAGGTAGATTTGCAAAGAATGACTGGAGTAACTATACACAGTCAAAT**<br>**GACTACTCATTCAAGTCTGCTTCACAGTTTGTTGAATGGGATCAGGTAACAGCATAC**<br>**TTGAACGGTGTTCTTGTATGGGGTAAAGAACCCGGTGGCAGTGTAGTACCATCAACA**<br>**CAGCCTGTAACAACACCACCTGCAACAACAAAACCACCTGCAACAACAAAACCACCT**<br>**GCAACAACAATACCGCCGTCAGATGATCCG**AGCACA**CTGCAGGTTGATATTGGTAGC**<br>**ACCAGCGGTAAAGCAGGTAGCGTTGTTAGCGTTCCGATTACCTTTACCAATGTTCCG**<br>**AAAAGCGGTATTTATGCACTGAGCTTTCGTACCAATTTTGATCCGCAGAAAGTTACC**<br>**GTTGCAAGCATTGATGCAGGTAGCCTGATTGAAAATGCAAGCGATTTTACCACCTAC**	2 508	89 248

		**TACAATAACGAAAATGGCTTTGCCAGCATGACCTTTGAAGCACCGGTTGATCGTGCA**<br>**CGTATTATTGATAGTGATGGTGTTTTTGCCACCATCAACTTCAAAGTTAGCGATAGC**<br>**GCAAAAGTTGGCGAACTGTATAACATTACCACCAATAGCGCATATACCAGCTTCTAT**<br>**TATAGCGGCACCGATGAAATCAAAAACGTGGTGTATAACGATGGCAAAATTGAAGTT**<br>**ATTGCCAGTCCAACACCAACACAG**CCAACG**AGCAGTCCGGGTAACAAAATGAAAATT**<br>**CAGATCGGTGATGTGAAAGCCAATCAGGGTGATACCGTTATTGTTCCGATTACCTTT**<br>**AATGAAGTTCCGGTGATGGGTGTGAACAATTGTAATTTTACCCTGGCCTACGACAAA**<br>**AACATCATGGAATTCATTAGCGCAGATGCAGGCGATATTGTTACCCTGCCGATGGCA**<br>**AATTATTCCTATAACATGCCTTCAGATGGCCTGGTGAAATTCCTGTATAATGATCAG**<br>**GCACAGGGTGCCATGAGCATTAAAGAAGATGGCACCTTTGCCAACGTGAAGTTCAAA**<br>**ATCAAACAGAGCGCAGCCTTTGGTAAATATAGCGTTGGTATTAAAGCCATTGGTAGC**<br>**ATTAGTGCACTGAGCAATAGCAAACTGATTCCGATTGAAAGCATCTTCAAAGATGGC**<br>**AGCATTACCGTTACCAATACGCCGACGAACACG**ACGAGC**CCGAAAACCACCATTATT**<br>**GCAGGTAGCGCAGAAGCACCGCAGGGTAGCGATATTCAGGTTCCGGTTAAAATTGAA**<br>**AACGCCGATAAAGTGGGTAGCATTAATCTGATTCTGAGCTATCCGAATGTTCTGGAA**<br>**GTTGAAGATGTTCTGCAGGGTAGTCTGACCCAGAATAGCCTGTTTGATTATAACGTT**<br>**GAAGGCAACCAGATCAAAGTTGGTATTGCAGATAGTAATGGCATTAGCGGTGATGGC**<br>**AGCCTGTTTTATGTTAAATTTCGTGTGACCGGCAACGAAAAAGCAGAACAGGCAGAA**<br>**AATGTTAAAGGTAAACTGCGTGGTCTGGGCCAGCAGCTGAGCGAAATTACCCTGCGT**<br>**AATAGCCATGCACTGACACTGCAGGGCATTGAAATTTATGACATTGATGGCAATAGC**<br>**GTGAAAGTGGCAACAATTAATGGCACCTTTCGTATTGTGAGCCAAGAGGAAGCCGTG**<br>**GTTCCGAGCACC***CCGTCTCATCATCACCATCACCATAAGTAC****GGTAAGCCTATCCCT***<br>***AACCCTCTCCTCGGACTGGATTCTACG****CGTACCGGTCATCATCACCATCACCATTGA*		
FDP	GFP—<br>Li—<br>Doc-*Ac*	**ATGTCTAAAGGTGAAGAATTATTCACTGGTGTTGTCCCAATTTTGGTTGAATTAGAT**<br>**GGTGATGTTAATGGTCACAAATTTTCTGTCTCCGGTGAAGGTGAAGGTGATGCTACT**<br>**TACGGTAAATTGACCTTAAAATTTATTTGTACTACTGGTAAATTGCCAGTTCCATGG**<br>**CCAACCTTAGTCACTACTTTAGGTTATGGTTTGATGTGTTTTGCTAGATACCCAGAT**<br>**CATATGAAACAACATGACTTTTTCAAGTCTGCCATGCCAGAAGGTTATGTTCAAGAA**	3 687	38 044

		**AGAACTATTTTTTTCAAAGATGACGGTAACTACAAGACCAGAGCTGAAGTCAAGTTT**<br>**GAAGGTGATACCTTAGTTAATAGAATCGAATTAAAAGGTATTGATTTTAAAGAAGAT**<br>**GGTAACATTTTAGGTCACAAATTGGAATACAACTATAACTCTCACAATGTTTACATC**<br>**ATGGCTGACAAACAAAAGAATGGTATCAAAGTTAACTTCAAAATTAGACACAACATT**<br>**GAAGATGGTTCTGTTCAATTAGCTGACCATTATCAACAAAATACTCCAATTGGTGAT**<br>**GGTCCAGTCTTGTTACCAGACAACCATTACTTATCCTATCAATCTGCCTTATCCAAA**<br>**GATCCAAACGAAAAGAGGGACCACATGGTCTTGTTAGAATTTGTTACTGCTGCTGGT**<br>**ATTACCCATGGTATGGATGAATTGTACAAA**GGTTCA**GGCGGTGGCGGTAGCGGTGGC**<br>**GGTGGCTCTGGTGGTGGCGGTAGC**TCTGGT**CCGACACCGAAATTCATTTATGGTGAT**<br>**GTTGATGGTAATGGCAGCGTGCGTATTAATGATGCAGTTCTGATTCGTGATTATGTG**<br>**CTGGGCAAAATTAACGAATTCCCGTATGAATATGGTATGCTGGCAGCAGATGTGGAT**<br>**GGCAATGGTAGCATTAAAATCAATGATGCCGTGCTGGTGCGCGATTACGTTCTGGGT**<br>**AAAATCTTTCTGTTTCCGGTGGAAGAAAAAGAAGAA**AAGTATCATCATCACCATCAC<br>CATTAA	

Table S4: Overview of the primers used for verification of yeast transformation. Column one indicates the vector for which presence is checked. The second column indicates primer orientation. In column three and four, the primer sequence (presented in the 5'-3' direction) and corresponding hybridization site are given.

Vector	Primer	Sequence (5'-3')	Hybridization site
pVTD20	FW	GATCGGACTACTAGCAGCTG	GAL promotor
	RV	GTACAGTGGGAACAAAGTCG	MATα terminator
pVTD35	FW	ACATTGCCTGAATTGG	AOD terminator
	RV	CTAGTTATTGCTCAGC	T7 terminator

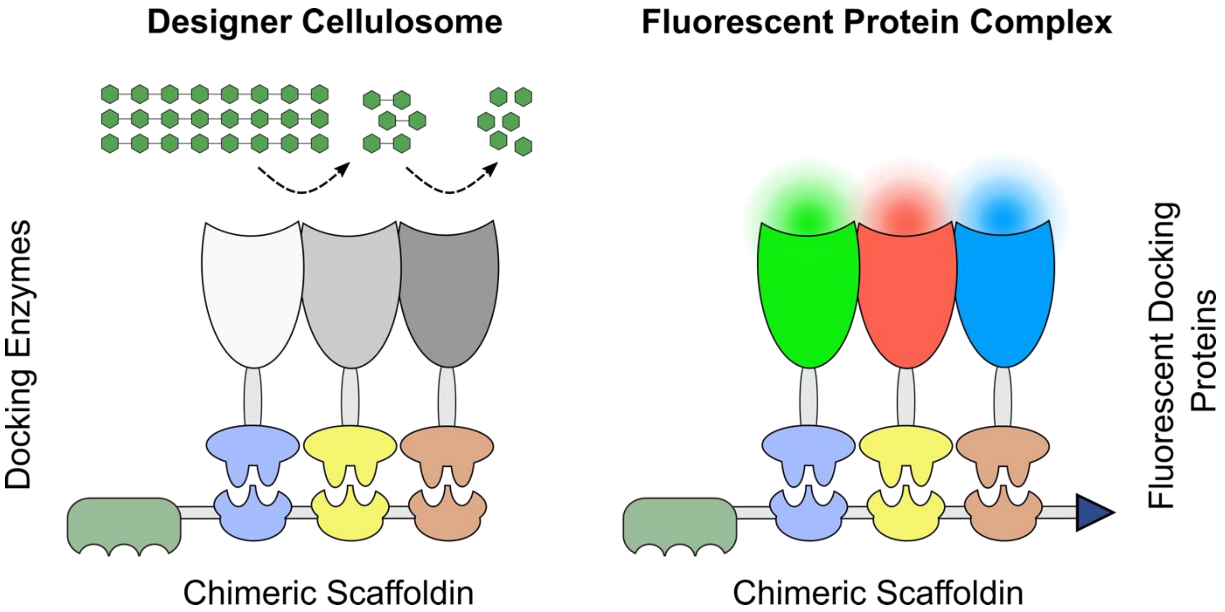

Figure S1: Comparison of a designer cellulosome and a fluorescent protein complex. In a DC, carbohydrate-active enzymes fused to a dockerin (DEs) are colocalized on a chimeric scaffoldin. As such, the DC executes enzymatic activity on a certain sugar polymer, in this example cellulolytic DEs are incorporated. A FPC follows the same architecture as a DC, colocalizing dockerin-fused proteins onto a chimeric scaffoldin. However, here the proteins exhibit fluorescence (FDPs) instead of enzymatic activity. Moreover, the scaffoldin contains an extra V5-tag which can be used for immunostaining and detection.

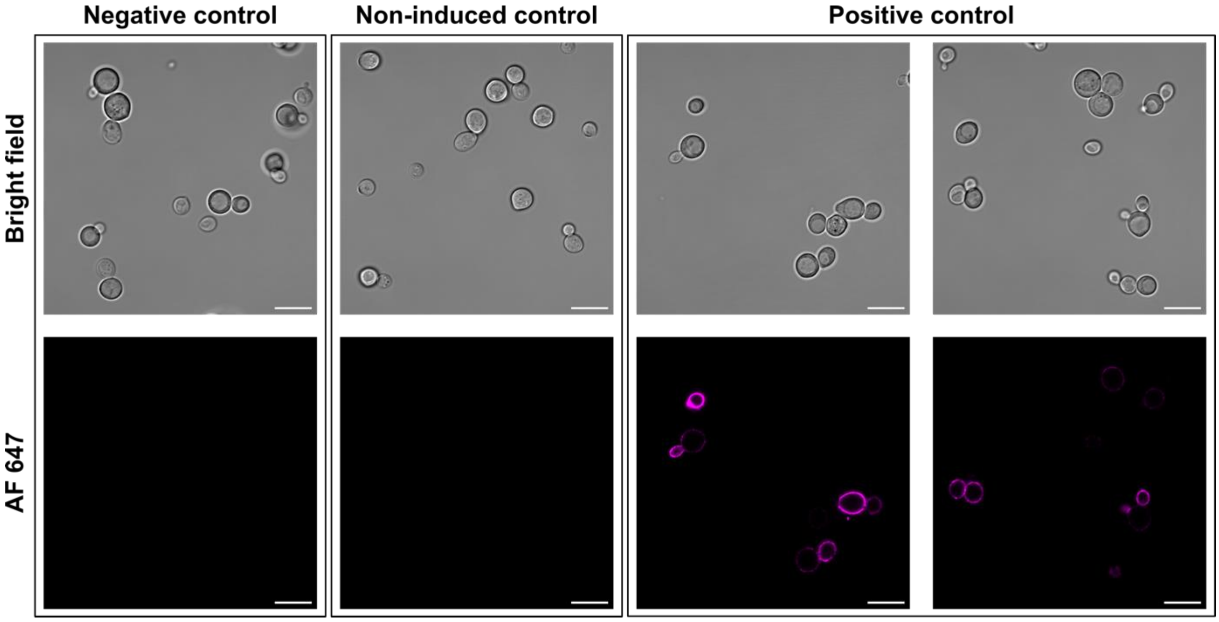

Figure S2: Examined controls regarding correct evaluation of antibody staining and scaffoldin display. First, the negative control implies scaffoldin-displaying yeast cells which are only stained with the secondary antibody, confirming that this antibody does not show aspecific binding. Second, a scaffoldin non-induced control was considered to exclude leaky expression of the scaffoldin. As no fluorescent signal was detected, this data corresponds to the FC analysis. Last, the positive control analyzed scaffoldin-induced yeast cells, stained with both antibodies. These images confirm successful scaffoldin display and staining on the yeast surface. Scale bars correspond to 10 µm.

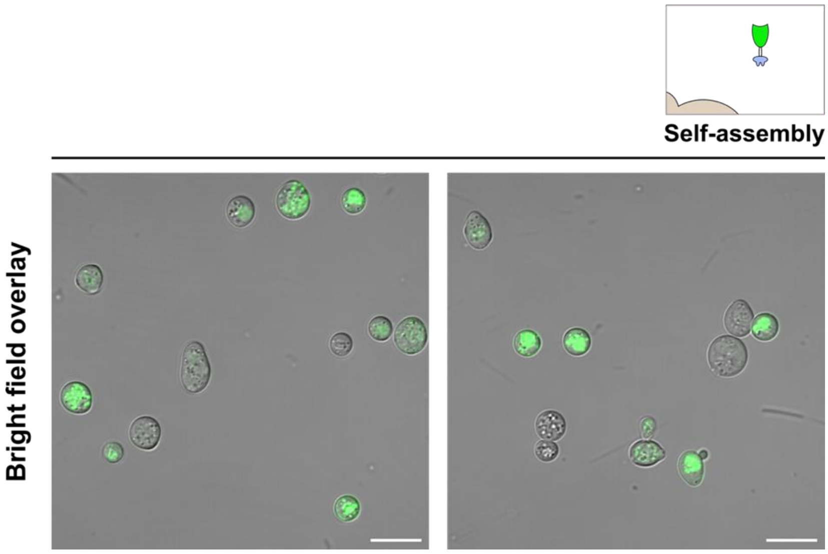

Figure S3: Scaffoldin non-induced control of FPC self-assembling yeast cells. The presented images are an overlay of the GFP and AF 647 signal on brightfield images. Scale bars correspond to 10 µm.

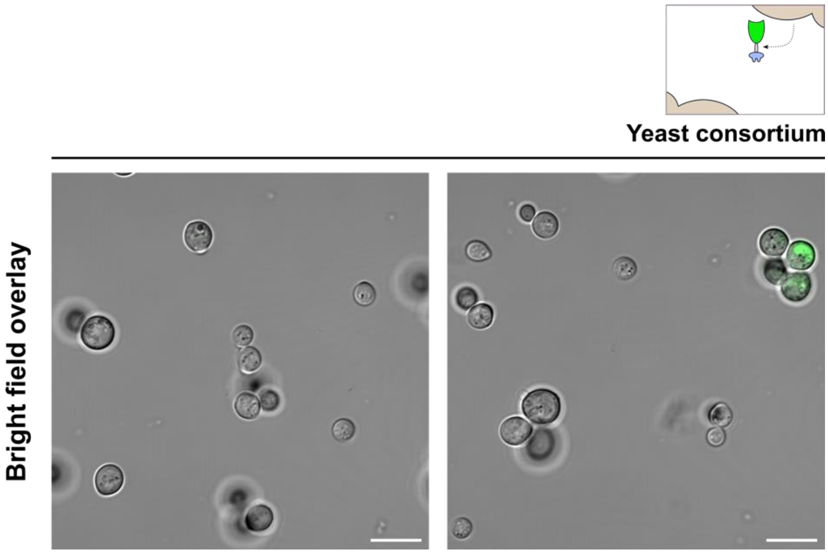

Figure S4: Scaffoldin non-induced control of yeast cells in a consortium. The presented images are an overlay of the GFP and AF 647 signal on brightfield images. Some green fluorescent cells can be detected. Most likely, these are remaining FDP-producing cells, inadvertently collected with the supernatant. Scale bars correspond to 10 µm.

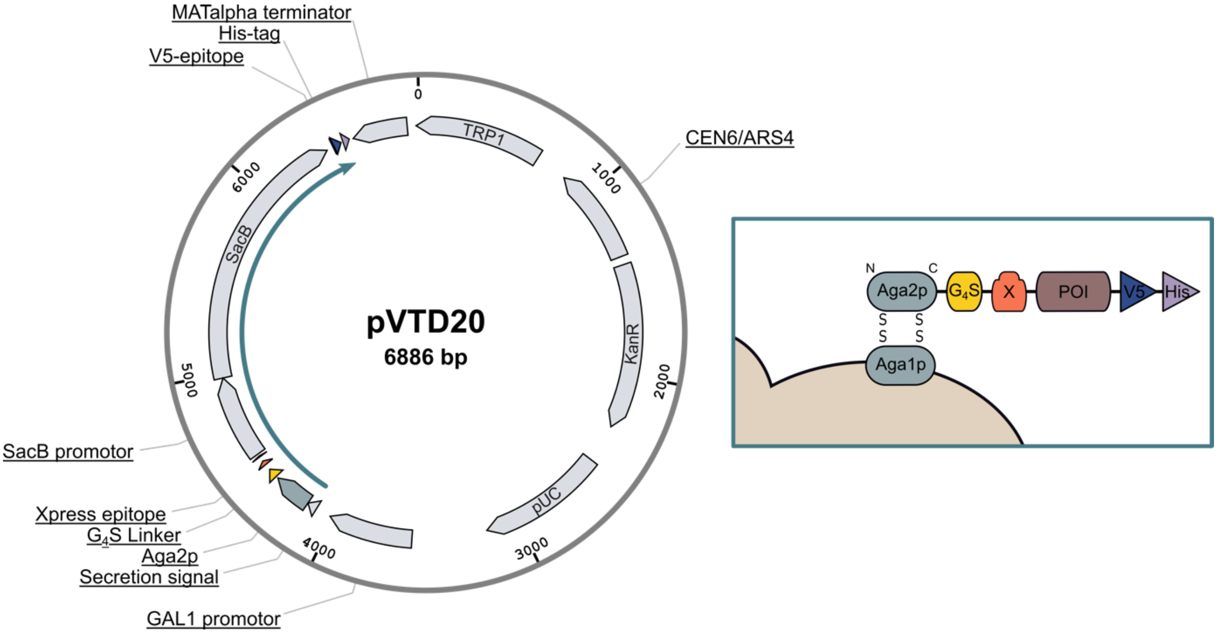

Figure S5: Graphical representation of the pVTD20 scaffoldin-display vector (left panel) and the corresponding displayed Aga2p-protein fusion (right panel). The pVTD20 vector contains two origins of replication, one for *E.coli* (pUC) and one for *S.cerevisiae* (CEN/ARS4), a kanamycin selection marker (KanR) and tryptophan auxotrophic marker (Trp). The vector carries a *sacB* cassette, peculiar to the VersaTile technique (1, 2). After a successful assembly reaction, the *sacB* cassette is replaced by the protein of interest's (POI) coding sequence, forming an Aga2p-POI fusion construct (indicated in the vector by a blue arrow). The corresponding Aga2p-protein fusion is shown in the right blue panel (depicted in the N- to C-terminal orientation) and contains an Aga2p subunit (blue gray), G_4S linker (yellow), Xpress-epitope (orange), POI (brown), V5-epitope (dark blue) and His-tag (purple). Expression of the Aga2p-protein fusion is regulated by the GAL1 promotor and MATα terminator. A secretion signal enables secretion of the Aga2p-protein fusion. Yeast cells containing the pVTD20 vector (Trp^+) can be selected by culturing them in tryptophan-deficient medium.

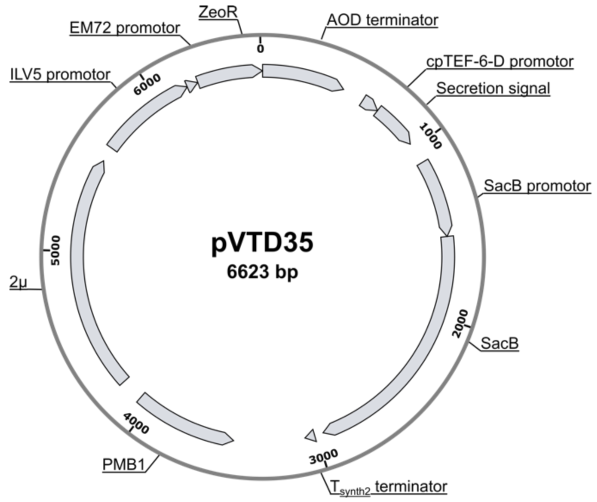

Figure S6: Graphical representation of the pVTD35 FDP-secretion vector. The pVTD35 vector contains a bacterial (PMB1) and yeast (2µ) origin of replication. The zeocin resistance open reading frame is provided with a bacterial (ILV5) and yeast (EM72) promotor and a yeast (AOD) terminator. The vector carries a *sacB* cassette, peculiar to the VersaTile technique (1, 2). After a successful assembly reaction, the *sacB* cassette is replaced by the POI's coding sequence. The assembled coding sequence is consequently preceded by a constitutive promotor, selected from the semi-synthetic yeast promotor library created by Decoene et al. (3), and the α-factor signal peptide DNA sequence (4). Downstream of the coding sequence, the short synthetic terminator T_{synth2} selected from the terminator set constructed by Curran et al. is present (5).

References

1. Vanderstraeten J, da Fonseca MJM, De Groote P, Grimon D, Gerstmans H, Kahn A, et al. Combinatorial assembly and optimisation of designer cellulosomes: a galactomannan case study. Biotechnol biofuels bioprod. 2022;15(1):60.
2. Gerstmans H, Grimon D, Gutierrez D, Lood C, Rodriguez A, van Noort V, et al. A VersaTile-driven platform for rapid hit-to-lead development of engineered lysins. Sci Adv. 2020;6(23):eaaz1136.
3. Decoene T, De Maeseneire SL, De Mey M. Modulating transcription through development of semi-synthetic yeast core promoters. Plos One. 2019;14(11):e0224476.
4. Aza P, Molpeceres G, de Salas F, Camarero S. Design of an improved universal signal peptide based on the α-factor mating secretion signal for enzyme production in yeast. Cell Mol Life Sci. 2021;78(7):3691-707.
5. Curran KA, Morse NJ, Markham KA, Wagman AM, Gupta A, Alper HS. Short synthetic terminators for improved heterologous gene expression in yeast. ACS Synth Biol. 2015;4(7):824-32.
