# Supplementary material for: Single-cell analysis of yeast surface display for designer cellulosome applications using a fluorescent protein complex
Source: Microbiol Spectr. 2025 Aug 12;13(9):e00750-25. doi: 10.1128/spectrum.00750-25 (PMC12403707; doi:10.1128/spectrum.00750-25)
Supplement: Supplemental movies legend — Legends for Movies S1 and S2. [file spectrum.00750-25-s0002.docx]

**Movie S1.mp4:** **3D visualization of a budding yeast cell performing FPC self-assembly.** This visualization shows that FDP production (green) and scaffoldin display (prink) are divided over the mother and daughter cell, respectively.

**Movie S2.mp4:** **3D visualization of two budding yeast cells of the synthetic yeast consortium.** FPC display is detected on the surface of the cells, as both pink and green fluorescent signal dedicated to the presence of the scaffoldin and FDP, respectively, can be observed. Note that the fluorescent signal is the strongest at the surface of newly emerging buds
